# Supplementary material for: Species and Strain Variability among Sarcina Isolates from Diverse Mammalian Hosts
Source: Animals (Basel). 2023 May 3;13(9):1529. doi: 10.3390/ani13091529 (PMC10177144; doi:10.3390/ani13091529)
Supplement: Supplementary file 1 [file animals-13-01529-s001.zip › Table S1_Animals_proof version.pdf]

Table S1. Positions of six genes within the genomes of nine bacterial strains used for primer pair construction. The NCBI database (https://www.ncbi.nlm.nih.gov/nuccore/) has been used for these purposes.

Strains	*ileS* (position)	*pheT* (position)	*pyrG* (position)	*rplB* (position)	*rplC* (position)	*rpsC* (position)
Clostridium carnis NCTC 10913^T	UYIN01000001 (1153251-1156364)	NZUYIN01000006 (c73540-71162)	UYIN01000001 (1274954-1276567)	NZUYIN01000020 (183618-184448)	NZUYIN01000020 (181987-182616)	NZUYIN01000020 (185167-185832)
C. celatum DSM 1785^T	AMEZ01000002 (28127-31237)	NZKB291681 (20477-22855)	AMEZ01000033 (29267-30865)	NZKB291623 (2861-3694)	NZKB291623 (1232-1861)	NZKB291623 (4422-5090)
C. disporicum 2789STDY5834855	CYZV01000005 (c59684-56574)	CYZV01000034 (c13255-10877)	CYZV01000044 (c27140-25536)	CYZV01000028 (c29517-28684)	CYZV01000028 (c31146-30517)	CYZV01000028 (c27957-27289)
C. sartagoforme AAU1	ASRV01000091 (c38061-34951)	ASRV01000086 (7001-9379)	ASRV01000213 (c4957-3356)	ASRV01000021 (c18384-17551)	ASRV01000021 (c20013-19384)	ASRV01000021 (c16821-16153)
C. septicum P1044	NZFLTT01000006 (5620-8730)	NZFLTT01000049 (c4309-1931)	NZFLTT01000017 (40563-42170)	NZFLTT01000042 (2633-3466)	NZFLTT01000042 (1000-1629)	NZFLTT01000042 (4196-4864)
C. tertium src5	NZOAOE01000010 (30231-33341)	NZOAOE01000002 (184806-187184)	NZOAOE01000024 (42450-44051)	NZOAOE01000018 (2787-3620)	NZOAOE01000018 (1158-1787)	NZOAOE01000018 (4350-5018)
Sarcina ventriculi NCTC 12966^T	UAUL01000007 (c267254-264144)	NZUAUL01000008 (37282-39660)	UAUL01000009 (19794-21398)	NZUAUL01000007 (c141122-140289)	NZUAUL01000007 (c142746-142117)	NZUAUL01000007 (c139552-138887)
S. ventriculi 14	NZBCMV01000019 (c60031-56921)	NZBCMV01000024 (10607-12985)	NZBCMV01000005 (c11072-9468)	NZBCMV01000016 (2473-3306)	NZBCMV01000016 (849-1478)	NZBCMV01000016 (4043-4708)
S. ventriculi 17	NZBCMW01000010 (c60041-56931)	NZBCMW01000003 (c374642-372264)	NZBCMW01000007 (17112-18716)	NZBCMW01000017 (2483-3316)	NZBCMW01000017 (859-1488)	NZBCMW01000017 (4053-4718)

Footnotes: NCTC - National Collection of Type Cultures, UK; DSM - German Collection of Microorganisms and Cell Cultures, c - complementary DNA sequence
