# Supplementary material for: Species and Strain Variability among Sarcina Isolates from Diverse Mammalian Hosts
Source: Animals (Basel). 2023 May 3;13(9):1529. doi: 10.3390/ani13091529 (PMC10177144; doi:10.3390/ani13091529)
Supplement: Supplementary file 1 [file animals-13-01529-s001.zip › Table S2_Animals_proof version.pdf]

Table S2. List of animal hosts cultured to detect *Sarcina*-like bacteria. Due to the large species and breed variability, there is a more detailed list of monkey and dog samples.

ANIMAL HOST		
**Primate sample**	**Primate species**	***Sarcina* spp.**
PR1	Common marmoset	✗
PR2	Common marmoset	✓
PR3	White-faced saki	✗
PR4	Emperor tamarin	✗
PR5	Moustached tamarin	✗
PR6	Brown-mantled tamarin	✗
PR7	Red-handed tamarin	✗
PR8	Red-handed tamarin	✗
PR9	Emperor tamarin	✗
PR10	Silvery marmoset	✗
PR11	Silvery marmoset	✗
PR15	Silvery marmoset	✗
PR16	Emperor tamarin	✗
PR17	Emperor tamarin	✗
PR18	Chimpanzee	✗
PR19	Northern white-cheeked gibbon	✗
PR20	Golden-bellied mangabey	✗
PR21	Diana monkey	✗
PR22	Lion-tailed macaque	✗
PR23	Hamadryas baboon	✗
PR24	Pygmy marmoset	✗
PR26	Cotton-top tamarin	✗
PR27	Golden lion tamarin	✗
PR28	Common marmoset	✗
PR29	Patas monkey	✓
PR30	Goeldi's marmoset	✗
PR31	White-headed marmoset	✗
PR32	White-headed marmoset	✗
PR33	Moustached tamarin	✗
PR34	Patas monkey	✗
PR35	Silvery marmoset	✗
PR36	Campbell's Mona monkey	✓
PR37	Putty-nosed monkey	✓
PR38	Northern Talapoin monkey	✓
PR39	De Brazza´s monkey	✓
PR40	Northern white-cheeked gibbon	✓
PR41	Chimpanzee	✗
PR42	Chimpanzee	✗
PR43	Chimpanzee	✗
PR44	Chimpanzee	✗

PR45	Patas monkey	✓
PR46	Yellow-cheeked crested gibbon	✓
PR47	Yellow-cheeked crested gibbon	✓
PR51	Yellow-cheeked crested gibbon	✓
PR52	Vervet monkey	✓
PR55	Hamlyn's monkey	✓
PR56	Roloway monkey	✓
PR57	Lesser spot-nosed monkey	✓
PR58	Yellow-cheeked crested gibbon	✗
PR59	Northern white-cheeked gibbon	✓
PR60	Northern white-cheeked gibbon	✓
PR61	Golden lion tamarin	✗
PR12	Ring-tailed lemur	✓
PR13	Ring-tailed lemur	✓
PR14	Ring-tailed lemur	✓
PR48	Ring-tailed lemur	✓
SIAM1-PR62	Gibbon siamang	✓
SIAM2-PR63	Gibbon siamang	✓
SIAM3-PR64	Gibbon siamang	✓
GLT-A	Golden lion tamarin	✗
GLT-B	Golden lion tamarin	✗
GLT-C	Golden lion tamarin	✗
GLT-D	Golden lion tamarin	✓
GLT-E	Golden lion tamarin	✗
GLT-F	Golden lion tamarin	✗
**Dog sample**	**Dog breed**	**Sarcina spp.**
VB1	German shepherd dog	✗
VB2	German shepherd dog	✗
VB3	German shepherd dog	✗
VB4	Golden retriever	✗
VB5	Samoyed	✗
VB6	German shepherd dog	✗
VB7	German shepherd dog	✗
VB8	German shepherd dog	✗
VB9	German shepherd dog	✗
VB10	Czechoslovakian wolfdog	✗
VB11	Crossbreed dog	✗
VB12	German shepherd dog	✗
VB13	Swiss shepherd	✗
VB14	German shepherd dog	✗
VB15	Labrador retriever	✗
VB16	Foxterier	✗
VB17	Belgian shepherd	✗
VB18	Czechoslovakian wolfdog	✗
VB19	Swiss shepherd	✗
VB20	Crossbreed dog	✗

VB21	Crossbreed dog	✗
VB22	German shepherd dog	✗
VB23	German shepherd dog	✗
VB24	Belgian shepherd	✗
VB25	Havanese	✗
VB26	Havanese	✗
VB27	Havanese	✗
VB28	Crossbreed dog	✗
VB29	Crossbreed dog	✗
VB30	Crossbreed dog	✗
VB31	Crossbreed dog	✗
VB32	Golden retriever	✗
VB33	German shepherd dog	✗
VB34	German shepherd dog	✗
VB35	German shepherd dog	✗
VB36	German shepherd dog	✗
VB37	German shepherd dog	✗
VB38	German shepherd dog	✗
VB39	German shepherd dog	✗
VB40	German shepherd dog	✗
VB41	German shepherd dog	✗
VB42	German shepherd dog	✗
VB43	German shepherd dog	✗
VB44	German shepherd dog	✗
VB45	German shepherd dog	✗
VB46	German shepherd dog	✗
VB47	German shepherd dog	✗
VB48	German shepherd dog	✗
VB49	German shepherd dog	✗
KK1	Border collie	✗
KK2	Border collie	✗
KK3	Border collie	✗
KK4	Border collie	✗
KK5	Border collie	✗
KK6	Border collie	✗
KK7	Border collie	✗
KK8	Cavalier king Charles spaniel	✗
KK9	Border collie	✗
KK10	Border collie	✗
KK11	Not known	✗
KK12	Border collie	✗
KK13	American staffordshire terrier	✓
KK14	Crossbreed dog	✗
KK15	Crossbreed dog	✗
KK16	Crossbreed dog	✗
KK17	Whippet	✗

KK18	Basenji	✗
KK19	Crossbreed dog	✗
KK20	Crossbreed dog	✗
KK21	Border collie	✗
**Elephant sample**	**Species**	**Sarcina spp.**
S1-8 (*n*=8)	Asian elephant	✓
K, D (*n*=2)	Asian elephant	✓
**Rhinoceros sample**	**Species**	**Sarcina spp.**
RH1-2 (*n*=2)	Eastern black rhinoceros	✓
**Calf sample**	**Breed**	**Sarcina spp.**
CA (*n*=48)	Holstein-Friesian calf	✗
CA (*n*=2)	Holstein-Friesian calf	✓

Footnotes: ✓ - detected, ✗ - not detected, PR1-48 – primate samples [17], other PR & GLT (unpublished data), VB – dog samples [23], KK – dog samples (unpublished data), S – elephant samples (unpublished data, Zoo Liberec), K, D – elephant samples (unpublished, Zoo Ústí nad Labem), RH – rhinoceros sample (unpublished, Safari park Dvůr Králové), CA – calf samples (unpublished, farm Vražkov).
