# Supplementary material for: Species and Strain Variability among Sarcina Isolates from Diverse Mammalian Hosts
Source: Animals (Basel). 2023 May 3;13(9):1529. doi: 10.3390/ani13091529 (PMC10177144; doi:10.3390/ani13091529)
Supplement: Supplementary file 1 [file animals-13-01529-s001.zip › Table S3_Animals_proof version.pdf]

Table S3. The NCBI accession numbers of genes used for phylogenetic reconstructions and the calculation of pairwise identity (in the study used *Sarcina* spp. isolates and their obtained NCBI accession numbers are marked in bold).

Strains	*ileS (position)*	*pheT* (position)	*pyrG* (position)	*rplB* (position)	*rplC* (position)	*rpsC* (position)	16S rRNA (position)
Clostridium butyricum DSM 10702^T	CP040626 (3838109-3841037)	CP040626 (1549315-1551693)	CP040626 (3450461-3452068)	CP040626 (3708046-3708876)	CP040626 (3709883-3710512)	CP040626 (3706662-3707330)	NR113244
C. carnis NCTC 10913^T	UYIN01000001 (1153251-1156364)	NZUYIN01000006 (c73540-71162)	UYIN01000001 (1274954-1276567)	UYIN01000020 (183618-184448)	UYIN01000020 (181987-182616)	UYIN01000020 (185167-185832)	UYIN01000001 (c823776-822274)
C. celatum DSM 1785^T	KB291597 (28127-31237)	NZKB291681 (20477-22855)	NZKB291625 (29267-30865)	NZKB291623 (2861-3694)	NZKB291623 (1232-1861)	NZKB291623 (4422-5090)	NZKB291630 (1-1430)
C. chauvoei DSM 7528^T	CP018624 (c2523240-2520130)	CP018624 (979516-981894)	CP018624 (164148-165754)	CP018624 (c2374593-2373760)	CP018624 (c2376226-2375597)	CP018624 (c2373030-2372362)	CP018624 (121222-122735)
C. intestinale DSM 6191^T	NZFQXU01000008 (195030-198140)	NZFQXU01000007 (c207130-204752)	NZFQXU01000007 (c30086-28479)	NZFQXU01000011 (3021-3854)	NZFQXU01000011 (1392-2021)	NZFQXU01000011 (4581-5246)	NZFQXU01000002 (1-1415)
C. isatidis DSM 15098^T	CP016786 (77419-80535)	CP016786 (805456-807796)	CP016786 (2585438-2587011)	CP016786 (253228-254061)	CP016786 (251601-252230)	CP016786 (254786-255457)	CP016786 (9316-10836)
C. perfringens ATCC 13124^T	CP000246 (3131476-3134386)	CP000246 (c2383355-2380974)	CP000246 (2742022-2743592)	CP000246 (2972427-2973260)	CP000246 (2974247-2974876)	CP000246 (2971023-2971691)	NR121697
C. scatologenes DSM 25775^T	CP009933 (c4315282-4312178)	CP009933 (5160306-5162649)	CP009933 (4249831-4251414)	CP009933 (4636370-4637197)	CP009933 (4634742-4635371)	CP009933 (4637918-4638589)	CP009933 (4591775-4593274)
C. septicum DSM 7534^T	CP023671 (1850871-1853893)	CP023671 (3173739-3176079)	CP023671 (2208761-2210368)	CP023671 (2011697-2012520)	CP023671 (2010064-2010693)	CP023671 (2013261-2013920)	AB971813
C. tarantellae DSM 3997^T	NZWHJC01000341 (c3249-139)	NZWHJC01000015 (c5308-2927)	NZWHJC01000033 (18162-19769)	NZWHJC01000022 (3494-4327)	NZWHJC01000022 (1869-2498)	NZWHJC01000022 (5069-5734)	NZWHJC01000270 (82-1591)
C. tertium DSM 2485^T	NZJAGGJY010000004 (c97853-94743)	NZJAGGJY010000007 (c53080-50702)	NZJAGGJY010000021 (42455-44056)	NZJAGGJY010000005 (c183396-182563)	NZJAGGJY010000005 (c185025-184396)	NZJAGGJY010000005 (c181833-181165)	Y18174
***Sarcina maxima* DSM 316^T**	**OK032175**	**OK032219**	**OK032245**	**OK032271**	**OK032297**	**OK032323**	**NR026147**
***S. maxima* 5a**	**OK032177**	**OK032221**	**OK032247**	**OK032273**	**OK032299**	**OK032325**	**MZ970332**
***S. maxima* 7**	**OK032200**	**OK032244**	**OK032270**	**OK032296**	**OK032322**	**OK032348**	**MZ970355**
***S. ventriculi* D3-1**	**OK032199**	**OK032243**	**OK032269**	**OK032295**	**OK032321**	**OK032347**	**MZ970354**

***S. ventriculi* DSM 286^{T}**	**OK032176**	**OK032220**	**OK032246**	**OK032272**	**OK032298**	**OK032324**	**MZ970331**
S. ventriculi NCTC 12966^{T}	UAUL01000007 (c267254-264144)	NZUAUL01000008 (37282-39660)	NZUAUL01000009 (19794-21398)	NZUAUL01000007 (c141122-140289)	NZUAUL01000007 (c142746-142117)	NZUAUL01000007 (c139552-138887)	NZUAUL01000001 (c554186-552677)
***S. ventriculi* Sa2**	**OK032195**	**OK032239**	**OK032265**	**OK032291**	**OK032317**	**OK032343**	**MZ970350**
***S. ventriculi* SIAM 3/5c**	**OK032196**	**OK032240**	**OK032266**	**OK032292**	**OK032318**	**OK032344**	**MZ970351**
S. ventriculi 14	NZBCMV01000019 (c60031-56921)	NZBCMV01000024 (10607-12985)	NZBCMV01000005 (c11072-9468)	NZBCMV01000016 (2473-3306)	NZBCMV01000016 (849-1478)	NZBCMV01000016 (4043-4708)	LC101491
S. ventriculi 17	NZBCMW01000010 (c60041-56931)	NZBCMW01000003 (c374642-372264)	NZBCMW01000007 (17112-18716)	NZBCMW01000017 (2483-3316)	NZBCMW01000017 (859-1488)	NZBCMW01000017 (4053-4718)	LC101492
***S. ventriculi* 12/4b**	**OK032197**	**OK032241**	**OK032267**	**OK032293**	**OK032319**	**OK032345**	**MZ970352**
***S. ventriculi* 13/5a**	**OK032198**	**OK032242**	**OK032268**	**OK032294**	**OK032320**	**OK032346**	**MZ970353**
***S. ventriculi* 39/7a**	**OK032178**	**OK032222**	**OK032248**	**OK032274**	**OK032300**	**OK032326**	**MZ970333**
***S. ventriculi* 39/7b**	**OK032179**	**OK032223**	**OK032249**	**OK032275**	**OK032301**	**OK032327**	**MZ970334**
***S. ventriculi* 46/5b**	**OK032180**	**OK032224**	**OK032250**	**OK032276**	**OK032302**	**OK032328**	**MZ970335**
***S. ventriculi* 47/5b**	**OK032181**	**OK032225**	**OK032251**	**OK032277**	**OK032303**	**OK032329**	**MZ970336**
***S. ventriculi* 48/3**	**OK032182**	**OK032226**	**OK032252**	**OK032278**	**OK032304**	**OK032330**	**MZ970337**
***S. ventriculi* 51/4a**	**OK032183**	**OK032227**	**OK032253**	**OK032279**	**OK032305**	**OK032331**	**MZ970338**
***S. ventriculi* 51/4c**	**OK032184**	**OK032228**	**OK032254**	**OK032280**	**OK032306**	**OK032332**	**MZ970339**
***S. ventriculi* 52/3c**	**OK032185**	**OK032229**	**OK032255**	**OK032281**	**OK032307**	**OK032333**	**MZ970340**
***S. ventriculi* 60/3c**	**OK032186**	**OK032230**	**OK032256**	**OK032282**	**OK032308**	**OK032334**	**MZ970341**
Candidatus Sarcina troglotydae JB2	CP051754 (2211988-2214898)	CP051754 (1703988-1706366)	CP051754 (1949002-1950564)	CP051754 (2088199-2089022)	CP051754 (2090017-2090646)	CP051754 (2086795-2087451)	CP051754 (9441-10960)
***Sarcina* sp. D3/3C**	**OK032187**	**OK032231**	**OK032257**	**OK032283**	**OK032309**	**OK032335**	**MZ970342**
***Sarcina* sp. K1/7A**	**OK032188**	**OK032232**	**OK032258**	**OK032284**	**OK032310**	**OK032336**	**MZ970343**
***Sarcina* sp. K3/7B**	**OK032189**	**OK032233**	**OK032259**	**OK032285**	**OK032311**	**OK032337**	**MZ970344**

Sarcina sp. N13/4e	OK032190	OK032234	OK032260	OK032286	OK032312	OK032338	MZ970345
Sarcina sp. S1/3c	OK032191	OK032235	OK032261	OK032287	OK032313	OK032339	MZ970346
Sarcina sp. S2/2b	OK032192	OK032236	OK032262	OK032288	OK032314	OK032340	MZ970347
Sarcina sp. S8/2c	OK032193	OK032237	OK032263	OK032289	OK032315	OK032341	MZ970348
Sarcina sp. S10/2a	OK032194	OK032238	OK032264	OK032290	OK032316	OK032342	MZ970349
