# Supplementary figures and images for: Species and Strain Variability among Sarcina Isolates from Diverse Mammalian Hosts
Source: Animals (Basel). 2023 May 3;13(9):1529. doi: 10.3390/ani13091529 (PMC10177144; doi:10.3390/ani13091529)

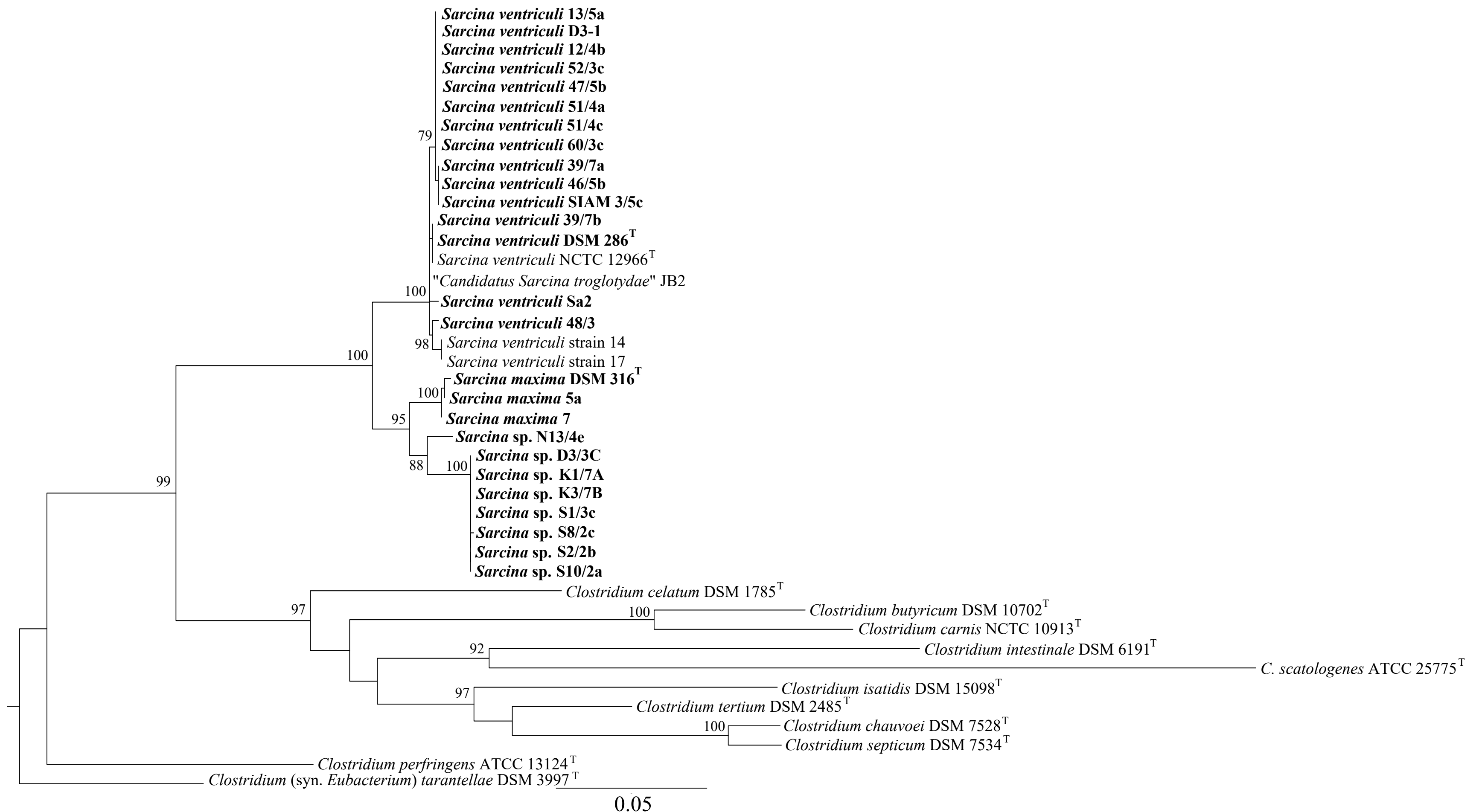

Supplement: Supplementary file 1 [file animals-13-01529-s001.zip › Figure S1_Animals_proof version.pdf]
